# Taxonomic position, antibiotic resistance and virulence factor production by *Stenotrophomonas* isolates from patients with cystic fibrosis and other chronic respiratory infections

**DOI:** 10.1186/s12866-022-02466-5

**Published:** 2022-05-12

**Authors:** Ad C. Fluit, Jumamurat R. Bayjanov, María Díez Aguilar, Rafael Cantón, Stuart Elborn, Michael M. Tunney, Jelle Scharringa, Barry J. Benaissa-Trouw, Miquel B. Ekkelenkamp

**Affiliations:** 1 Department of Medical Microbiology, University Medical Center Utrecht, PO Box 85500, 3508 GA Utrecht, the Netherlands; 2 Servicio de Microbiología, Hospital Universitario Ramón y Cajal and Instituto Ramón y Cajal de Investigación Sanitaria (IRYCIS), Madrid, Spain; 3 Red Española de Investigación en Patología Infecciosa (REIPI), Madrid, Spain; 4 Present Address: Servicio de Microbiología, Hospital Universitario La Princesa, Madrid, Spain; 5 Queen’s University Belfast, School of Pharmacy, Belfast, UK

**Keywords:** Stenotrophomonas, Cystic fibrosis, Respiratory infection, Taxonomy, Antibiotic resistance, Virulence

## Abstract

**Background:**

The potential pathogenic role of *Stenotrophomonas maltophilia* in lung disease and in particular in cystic fibrosis is unclear. To develop further understanding of the biology of this taxa, the taxonomic position, antibiotic resistance and virulence factors of *S. maltophilia* isolates from patients with chronic lung disease were studied.

**Results:**

A total of 111 isolates recovered between 2003 and 2016 from respiratory samples from patients in five different countries were included. Based on a cut-off of 95%, analysis of average nucleotide identity by BLAST (ANIb) showed that the 111 isolates identified as *S. maltophilia* by Matrix-assisted laser desorption/ionization time of flight mass spectrometry (MALDI-TOF/MS) belonged to *S. maltophilia* (*n* = 65), *S. pavanii* (n = 6) and 13 putative novel species (*n* = 40), which each included 1–5 isolates; these groupings coincided with the results of the 16S rDNA analysis, and the L1 and L2 ß-lactamase Neighbor-Joining phylogeny. Chromosomally encoded aminoglycoside resistance was identified in all *S. maltophilia* and *S. pavani* isolates, while acquired antibiotic resistance genes were present in only a few isolates. Nevertheless, phenotypic resistance levels against commonly used antibiotics, determined by standard broth microbroth dilution, were high. Although putative virulence genes were present in all isolates, the percentage of positive isolates varied. The Xps II secretion system responsible for the secretion of the StmPr1–3 proteases was mainly limited to isolates identified as *S. maltophilia* based on ANIb, but no correlation with phenotypic expression of protease activity was found. The RPF two-component quorum sensing system involved in virulence and antibiotic resistance expression has two main variants with one variant lacking 190 amino acids in the sensing region.

**Conclusions:**

The putative novel *Stenotrophomonas* species recovered from patient samples and identified by MALDI-TOF/MS as *S. maltophilia*, differed from *S. maltophilia* in resistance and virulence genes, and therefore possibly in pathogenicity. Revision of the *Stenotrophomonas* taxonomy is needed in order to reliably identify strains within the genus and elucidate the role of the different species in disease.

**Supplementary Information:**

The online version contains supplementary material available at 10.1186/s12866-022-02466-5.

## Background

*Stenotrophomonas* species are non-fermenting gram-negative rods. Currently sixteen species are recognized within the genus: *S. acidaminiphila*, *S. bentonitica*, *S. chelatiphaga*, *S. daejeonensis*, *S. ginsengisoli*, *S. humi*, *S. indicatrix*, *S. koreensis*, *S. lactitubi*, *S. maltophilia*, *S. nitrireducens*, *S. pavanii*, *S. pictorum*, *S. rhizophila*, *S. terrae*, *S. tumulicola*. To note that *S. africana* is no longer recognized as a species and has been added to *S. maltophilia* [1]. However, the taxonomy of the genus is not completely resolved. *S. maltophilia* forms a complex of which the isolates show considerable heterogeneity in genetic and phenotypic characteristics. Based on amplified fragment length polymorphism and DNA hybridizations 10 genogroups (#1–10) were defined [2]. This was expanded by the use of Multi-Locus Sequence Typing (MLST) with a representative of each genogroup and additional strains, which confirmed the ten genogroups and found five additional groups (A-E) [3]. Another MLST-based study added lineage (F) and genospecies Smc 1–4 and, e.g., genogroup #8 was shown to cluster with the *S. rhizophila* type strain [4]. In this scheme, *S. pavanii* was included within *S. maltophilia*.

Soils are an important reservoir for *Stenotrophomonas* species, but some species found in the environment may be opportunistic human pathogens, in particular *S. maltophilia*. Although *S. maltophilia* is considered a low-grade pathogen, it carries a large number of (putative) virulence factors [5]. The (putative) virulence factors include proteases (StmPr1/alkaline serine protease, StmPr2, StmPr3, StmPr4, gelatinase, elastase, and fibrinolysin/streptokinase), nucleases (DNase and 2 RNases), lipases (lipase, and phospholipase C and D) adhesion factors (SMF-1, TadE-like, giant cable pilus-like afimbrial adhesin, and type IV pilus machinery), siderophores, biofilm formation factors (phosphoglucomutase/phosphomannose bifunctional protein, glucose-1-phosphate thymylyltransferase, biofilm and swimming motility regulator, and Ax21 outer membrane protein), and a range of other factors (polysaccharide lyase, nitrate reductase, ankyrin repeat domain-containing protein, regulators RpfC and RpfF, hemagglutinin, esterase, hyaluronidase, heparinase, hemolysin, cytotoxin(s), and Xps type II secretion system). However, it cannot be excluded that some of these factors are part of the normal house-keeping processes.

*S. maltophilia* isolates usually exhibit high levels of phenotypic antibiotic resistance, due to an arsenal of intrinsic resistance genes and mutationally acquired resistance. *S. maltophilia* encodes several efflux pumps, of which at least six have been shown to expel a range of different antibiotics. Furthermore, *S. maltophilia* encodes the metallo-ß-lactamase L1 and the extended-spectrum ß-lactamase L2. L1 hydrolyzes carbapenems and other ß-lactam antibiotics with the exception of monobactams, whereas L2 is a serine hydrolase that acts as a cephalosporinase [6–9]. A chromosomally encoded *qnr* gene (*smqnr*) confers low-level fluoroquinolone resistance. Finally, acquisition of *sul1*, *sul2*, and *sul3* has been also described, resulting in sulfonamide resistance and contributing to co-trimoxazole resistance. Adegoke et al. recently published a review of *S. maltophilia* antibiotic resistance determinants [10].

Infections by *Stenotrophomonas* species are usually caused by species identified as *S. maltophilia*, and mostly involve the airways [11]. However, due to their high intrinsic antibiotic resistance, *Stenotrophomonas* species may be recovered as a colonizers in patients treated with long-term broad spectrum antibiotic therapy, which obscures their role in chronic lung disease [12]. This is further complicated by the heterogeneity within the *S. maltophilia* complex, which is not distinguished by current microbiological diagnostics.

The aim of the present study was to determine the taxonomic position of *S. maltophilia* isolates from persons with cystic fibrosis (CF) and patients with other chronic respiratory infections, and to characterize their antibiotic resistance genes and virulence factors.

## Results

### Genome characteristics

Sequencing and assembly of the whole genomes of the isolates resulted in an average of 181 contigs larger than 1000 bp, an average coverage of 81x, and a genome size of approximately 4.70 MB, indicating high quality of the sequences. The isolates belonged to 55 different Sequence Types (ST) including 16 novel types. Forty-three novel alleles were found: five for *atpD*, four for *gapA*, thirteen for *guaA*, seven for *mutM*, three for *nuoD*, six for *ppsA*, and five for *recA*. Twenty-one isolates belonged to ST5, nine to ST4 and to ST31, seven to the novel ST535, five to ST162, and four to ST26 (Table 1). Of other STs three isolates or less were found.

**Table 1:** Taxonomy, ST, isolate source, country of origin and resistance genes of the isolates

Order nr	ANIb	ST	Isolate source	Country	Resistance genes
543,227	A	89	CF	Northern Ireland	–
533,506	A	215	CF	the Netherlands	–
534,845	A	549	CF	Spain	–
534,836	B	130	CF	Spain	–
546,334	C	133	other	Northern Ireland	–
543,210	D	536	CF	Northern Ireland	–
533,529	D	337	CF	the Netherlands	–
534,834	D	337	CF	Spain	–
533,533	E	39	CF	the Netherlands	–
534,812	E	39	CF	Spain	–
534,818	E	39	CF	Spain	aac(6′)-Iz
542,535	E	39	CF	Northern Ireland	–
543,206	E	39	CF	Northern Ireland	–
543,208	E	39	CF	Northern Ireland	–
543,215	E	39	CF	Northern Ireland	–
543,217	E	39	CF	Northern Ireland	–
546,336	E	39	other	Northern Ireland	–
543,216	E	180	CF	Northern Ireland	–
542,523	F	535	CF	Northern Ireland	
542,525	F	535	CF	Northern Ireland	
542,527	F	535	CF	Northern Ireland	
542,530	F	535	CF	Northern Ireland	
543,225	F	535	CF	Northern Ireland	
547,141	F	535	CF	Northern Ireland	
548,978	F	535	other	Northern Ireland	
534,846	G	537	CF	Spain	–
543,222	H	79	CF	Northern Ireland	–
533,514	H	545	CF	the Netherlands	–
548,948	I	34	other	the Netherlands	–
534,842	J	548	CF	Spain	–
533,515	K	29	CF	the Netherlands	–
533,513	K	77	CF	the Netherlands	–
543,213	K	77	CF	Northern Ireland	–
548,945	K	219	other	Northern Ireland	–
533,531	K	224	CF	the Netherlands	–
534,799	K	538	CF	Spain	–
534,811	L	15	CF	Spain	–
542,542	L	15	CF	Northern Ireland	–
534,821	M	533	CF	Spain	aac(6′)-Ib3, ant(2″)-Ia, aac(6′)-Ib-cr
534,822	M	534	CF	Spain	aac(6′)-Ib3, ant(2″)-Ia, aac(6′)-Ib-cr
533,501	*S. maltophilia*	4	CF	the Netherlands	aph(3′)-IIc, aac(6′)-Iz
533,507	*S. maltophilia*	4	CF	the Netherlands	aph(3′)-IIc, aac(6′)-Iz
533,524	*S. maltophilia*	4	CF	the Netherlands	aph(3′)-IIc, aac(6′)-Iz
533,532	*S. maltophilia*	4	CF	the Netherlands	aph(3′)-IIc, aac(6′)-Iz
533,534	*S. maltophilia*	4	CF	the Netherlands	aph(3′)-IIc, aac(6′)-Iz
534,831	*S. maltophilia*	4	CF	Spain	aph(3′)-IIc, aac(6′)-Iz
534,838	*S. maltophilia*	4	CF	Spain	aph(3′)-IIc, aac(6′)-Iz
542,522	*S. maltophilia*	4	CF	Northern Ireland	aph(3′)-IIc, aac(6′)-Iz
548,946	*S. maltophilia*	4	CF	Northern Ireland	aph(3′)-IIc, aac(6′)-Iz
533,502	*S. maltophilia*	5	CF	the Netherlands	aph(3′)-IIc, aac(6′)-Iz, aph(3″)-Ib, aph(6)-Id
533,518	*S. maltophilia*	5	CF	the Netherlands	aph(3′)-IIc, aac(6′)-Iz
534,802	*S. maltophilia*	5	CF	Spain	aph(3′)-IIc, aac(6′)-Iz
534,804	*S. maltophilia*	5	CF	Spain	aph(3′)-IIc, aac(6′)-Iz, aph(6)-Id, aph(4)-Ia, aac(6′)-Ib-cr, aph(3″)-Ib, aac(3)-IV, aph(3′)-Ia, aac(6′)-Ib-Hangzhou, tet(G), sul1, cmx
534,807	*S. maltophilia*	5	CF	Spain	aph(3′)-IIc, aac(6′)-Iz
534,823	*S. maltophilia*	5	CF	Spain	aph(3′)-IIc, aac(6′)-Iz
534,825	*S. maltophilia*	5	CF	Spain	aph(3′)-IIc, aac(6′)-Iz
534,829	*S. maltophilia*	5	CF	Spain	aph(3′)-IIc, aac(6′)-Iz
534,839	*S. maltophilia*	5	CF	Spain	aph(3′)-IIc, aac(6′)-Iz
534,843	*S. maltophilia*	5	CF	Spain	aph(3′)-IIc, aac(6′)-Iz
540,831	*S. maltophilia*	5	CF	the Netherlands	aph(3′)-IIc, aac(6′)-Iz, aph(6)-Id, aac(6′)-Ib-cr, aph(3″)-Ib, aac(6′)-Ib3, ant(2″)-Ia, sul1
542,536	*S. maltophilia*	5	CF	Northern Ireland	aph(3′)-IIc, aac(6′)-Iz
542,538	*S. maltophilia*	5	CF	Northern Ireland	aph(3′)-IIc, aac(6′)-Iz
542,539	*S. maltophilia*	5	CF	Northern Ireland	aph(3′)-IIc, aac(6′)-Iz
543,203	*S. maltophilia*	5	CF	Northern Ireland	aph(3′)-IIc, aac(6′)-Iz
543,219	*S. maltophilia*	5	CF	Northern Ireland	aph(3′)-IIc, aac(6′)-Iz
546,340	*S. maltophilia*	5	CF	the Netherlands	aph(3′)-IIc, aac(6′)-Iz
546,343	*S. maltophilia*	5	CF	the Netherlands	aph(3′)-IIc, aac(6′)-Iz
547,144	*S. maltophilia*	5	CF	Northern Ireland	aph(3′)-IIc, aac(6′)-Iz
547,147	*S. maltophilia*	5	CF	Northern Ireland	aph(3′)-IIc, aac(6′)-Iz, aph(3″)-Ib, aph(6)-Id
548,947	*S. maltophilia*	5	CF	the Netherlands	aph(3′)-IIc, aac(6′)-Iz
533,517	*S. maltophilia*	8	CF	the Netherlands	aph(3′)-IIc
534,844	*S. maltophilia*	26	CF	Spain	aph(3′)-IIc
542,532	*S. maltophilia*	26	CF	Northern Ireland	aph(3′)-IIc
542,534	*S. maltophilia*	26	CF	Northern Ireland	aph(3′)-IIc
543,223	*S. maltophilia*	26	CF	Northern Ireland	aph(3′)-IIc
533,525	*S. maltophilia*	27	CF	the Netherlands	aph(3′)-IIc
533,509	*S. maltophilia*	31	CF	the Netherlands	aph(3′)-IIc
533,530	*S. maltophilia*	31	CF	the Netherlands	aph(3′)-IIc
546,338	*S. maltophilia*	31	CF	Northern Ireland	aph(3′)-IIc
534,806	*S. maltophilia*	84	CF	Spain	aph(3′)-Iic, aac(3)-IV, aph(3″)-Ib, aph(6)-Id, aph(4)-Ia, drfA15, tet(A), sul1
534,817	*S. maltophilia*	92	CF	Spain	aph(3′)-IIc, aac(6′)-Iz
534,840	*S. maltophilia*	146	CF	Spain	aph(3′)-IIc, aac(6′)-Iz
534,835	*S. maltophilia*	162	CF	Spain	aph(3′)-IIc
543,221	*S. maltophilia*	162	CF	Northern Ireland	aph(3′)-IIc
546,339	*S. maltophilia*	162	CF	the Netherlands	aph(3′)-IIc
547,143	*S. maltophilia*	162	CF	Northern Ireland	aph(3′)-IIc
548,949	*S. maltophilia*	162	CF	the Netherlands	aph(3′)-IIc
548,950	*S. maltophilia*	199	CF	the Netherlands	aph(3′)-IIc, aac(6′)-Iz
533,512	*S. maltophilia*	246	CF	the Netherlands	aph(3′)-IIc, aac(6′)-Iz
534,832	*S. maltophilia*	246	CF	Spain	aph(3′)-IIc, aac(6′)-Iz
543,220	*S. maltophilia*	531	CF	Northern Ireland	aph(3′)-IIc, aac(6′)-Iz
534,809	*S. maltophilia*	532	CF	Spain	aph(3′)-IIc, aac(6′)-Iz
533,510	*S. maltophilia*	539	CF	the Netherlands	aph(3′)-IIc
533,527	*S. maltophilia*	540	CF	the Netherlands	aph(3′)-IIc, aac(6′)-Iz
533,528	*S. maltophilia*	541	CF	the Netherlands	aph(3′)-IIc, aac(6′)-Iz
539,959	*S. maltophilia*	541	CF	the Netherlands	aph(3′)-IIc, aac(6′)-Iz
534,828	*S. maltophilia*	542	CF	Spain	aph(3′)-IIc, aac(6′)-Iz
534,808	*S. maltophilia*	543	CF	Spain	aph(3′)-IIc, aac(6′)-Iz
534,801	*S. maltophilia*	544	CF	Spain	aph(3′)-IIc, aac(6′)-Iz
543,211	*S. maltophilia*	546	CF	Northern Ireland	aph(3′)-IIc
534,805	*S. maltophilia*	547	CF	Spain	aph(3′)-IIc, aac(6′)-Iz
546,337	*S. maltophilia*	550	CF	Northern Ireland	aph(3′)-IIc, aac(6′)-Iz
546,342	*S. maltophilia*	551	CF	the Netherlands	aph(3′)-IIc, aac(6′)-Iz
547,148	*S. maltophilia*	539	other	Northern Ireland	aph(3′)-IIc
534,819	*S. maltophilia*	xx^a^	CF	Spain	aph(3′)-IIc, aac(6′)-Iz
547,145	*S. pavanii*	23	other	Northern Ireland	aac(6′)-Iak
534,815	*S. pavanii*	24	CF	Spain	aac(6′)-Iak
545,270	*S. pavanii*	220	other	USA	aac(6′)-Iak
534,800	*S. pavanii*	233	CF	Spain	aac(6′)-Iak
542,603	*S. pavanii*	233	CF	Australia	aac(6′)-Iak
533,516	*S. pavanii*	306	CF	the Netherlands	aac(6′)-Iak

^a^xx no ST available because allele mutM is partially deleted

Taxonomy, ST, isolate source, country of origin and resistance genes of the isolates used in this study

### Stenotrophomonas taxonomy

**Fig. 1:**
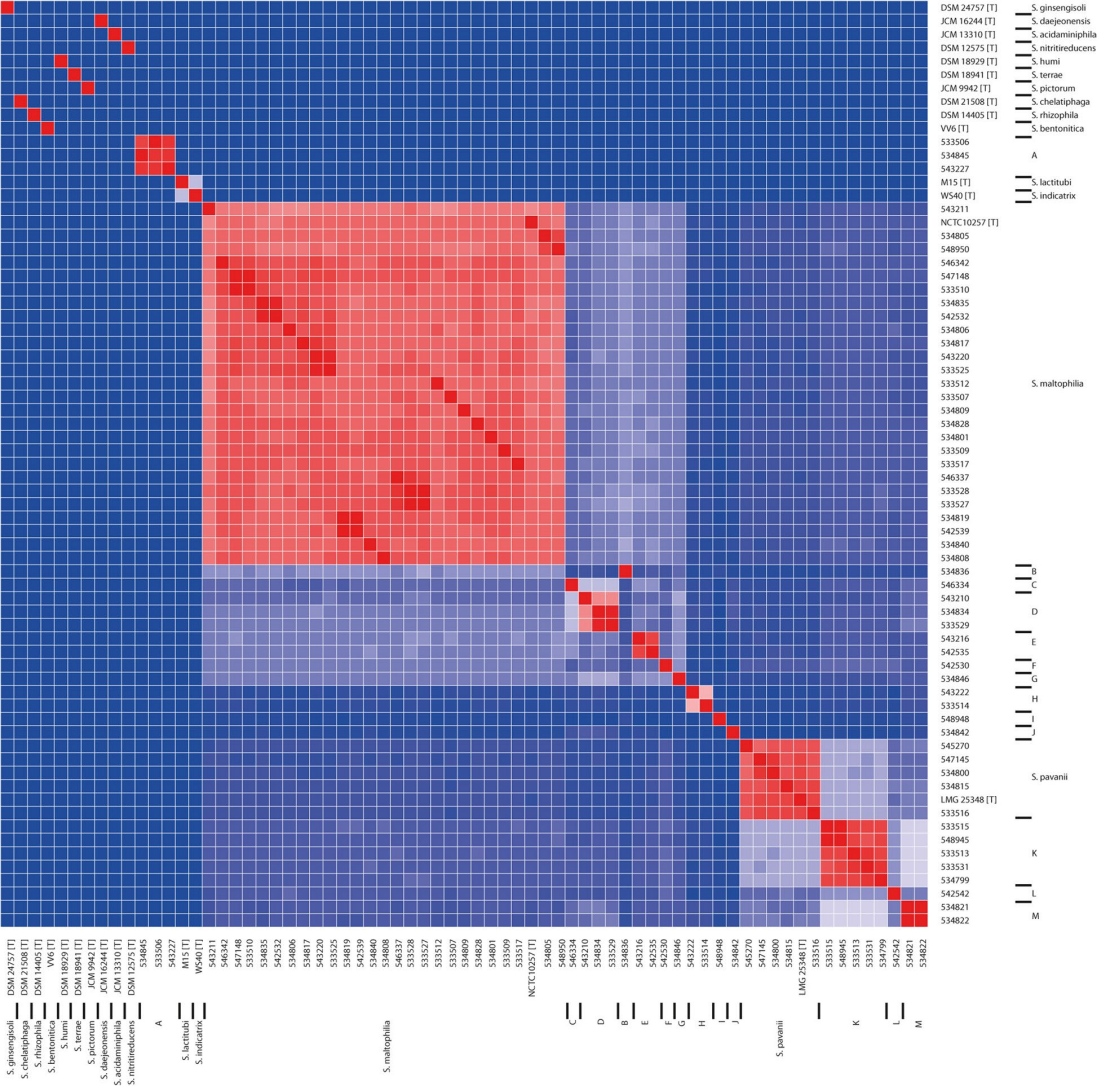
Heatmap based on percentage of ANIb for strains belonging to the different STs that were analyzed in this study (one isolate per ST was included) as well as available type strain sequences. The color bar shows the percentage of ANIb between any two strains starting from blue (0.8 or 80%) to red (1 or 100%); the blue/red cutoff is 0.94/0.95 (see Methods section for more details). Type strains are indicated by (T) behind the strain identification. The putative species (see text) are indicated at the right. For details about strains and the similarity between strains see Supplementary Fig. 1

To assess the taxonomic position of the *Stenotrophomonas* isolates an average nucleotide identity by BLAST (ANIb) was performed (Fig. 1 and Supplementary Fig. 1). Based on a cut-off of 95% identity for the boundary between species, the type strains of the different species were separated. However, also 13 groups of 1–5 isolates (indicated by a capital letter) may be considered as separate species when applying this cut-off. *S. maltophilia*, *S. pavanii*, and putative species B-M appeared to be more closely related to each other (identity = > 90%) than to other species within the genus (identity < 90%). Based on the ANIb analysis, only 65 of the 111 isolates (58.6%) initially identified as *S. maltophilia* by MALDI-TOF/MS could be confirmed as belonging to this species. The 46 other isolates would either be *S. pavani* (*n* = 6) or belong to the putative novel species B-M (*n* = 40).

**Fig. 2:**
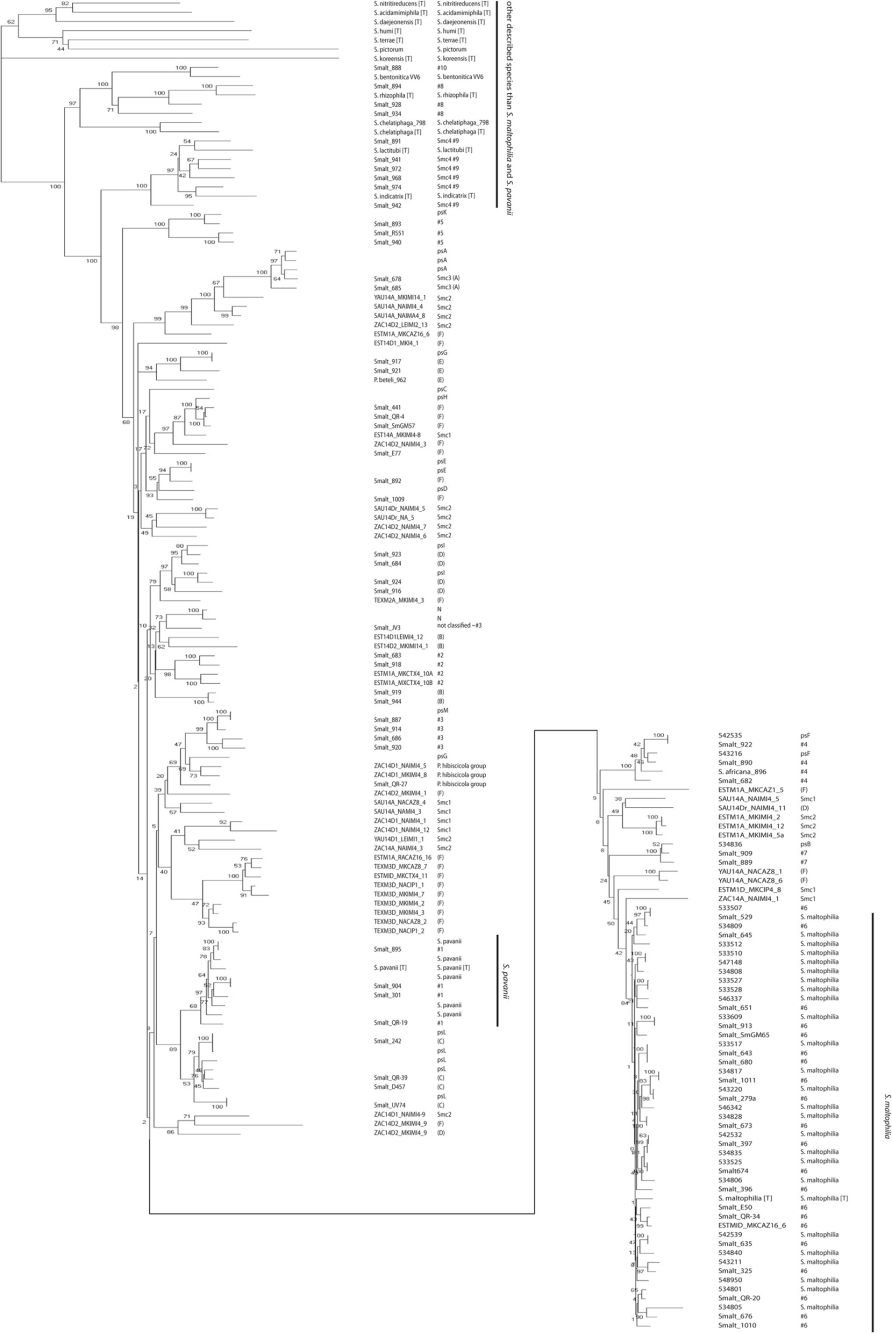
Evolutionary history of *Stenotrophomonas* strains based on concatenated alleles from the *S. maltophilia* MLST scheme (pubmlst.org). The figure combines data from Ochoa-Sánchez and Vinuesa and our study (Ochoa-Sánchez and Vinuesa, 2017). Lineages #1-#10 were defined by Hauben et al. (Hauben et al., 1999). Lineages (A) to (F) were defined by Kaiser et al. (Kaiser et al., 2009) and Ochoa-Sanchez et al. (Ochoa-Sanchez 2017) expanded this further. The putative species A-N from the ANIb analysis are indicated by ps and the letter. The evolutionary history was inferred using the Neighbor-Joining method (Saitou, et al., 1987). The optimal tree with the sum of branch length = 1.82770971 is shown. The percentage of replicate trees in which the associated taxa clustered together in the bootstrap test (500 replicates) are shown next to the branches (Felsenstein, 1985). The tree is drawn to scale, with branch lengths in the same units as those of the evolutionary distances used to infer the phylogenetic tree. The analysis involved 190 amino acid sequences. All ambiguous positions were removed for each sequence pair. There were a total of 3710 positions in the final dataset. Evolutionary analyses were conducted in MEGA X (Kumar et al., 2018). The left column denotes the isolates and strain identification. The right column the grouping. Type strains are indicated by (T) behind the name.

To assess the relationship of our isolates with the data of Ochoa-Sánchez and Vinuesa, a Neighbor-Joining tree of the concatenated MLST alleles was created with the STs in our collection, the dataset described by Ochoa-Sánchez and Vinuesa, and the available type strains [4]. Results showed that isolates previously identified based on MLST as lineages #1-#10 by Hauben et al., lineages (A)-(E) by Kaiser et al., and lineage (F) and genospecies Smc 1–4 by Ochoa-Sánchez and Vinuesa, as well as isolates that belonged to previously defined species other than *S. maltophilia* and *S. pavanii*, clustered in well-defined branches (Fig. 2) [2–4]. Lineage #8 isolates clustered with the *S. rhizophila* type strain and the lineage #10 isolate with the *S. bentonitica* type strain. The lineage #9 isolates clustered with both the *S. indicatrix* and the *S. lactitubi* type strains. Lineages #1 and #6 corresponded with *S. pavanii* and *S. maltophilia*, respectively (Fig. 2). Lineage #3 isolates clustered with the putative species L isolate; lineage #4 isolates with putative species E isolates; lineage #5 isolates with the putative species J isolate; lineage #7 isolates with the putative species B isolate. From the other lineages that have been defined, cluster lineage (C) isolates with putative species K isolates and Smc3 isolates clustered with putative species A isolates. Lineage (E) isolates cluster with the putative species I isolate. One of the (E) isolates has previously been named *Pseudomonas beteli*, which is now considered synonymous with *S. maltophilia*. Similarly, isolates previously named *Pseudomonas hibiscicola*, which is also considered synonymous with *S. maltophilia,* clustered with putative species F [1]. Isolates from some other lineages, e.g. #2 and (B) also clustered, but not with any of the known or putative species as determined by ANIb. However, the isolates previously grouped as (D), (F), Smc1 or Smc2 did not group together in the ST Neighbor-Joining phylogeny.

**Fig. 3:**
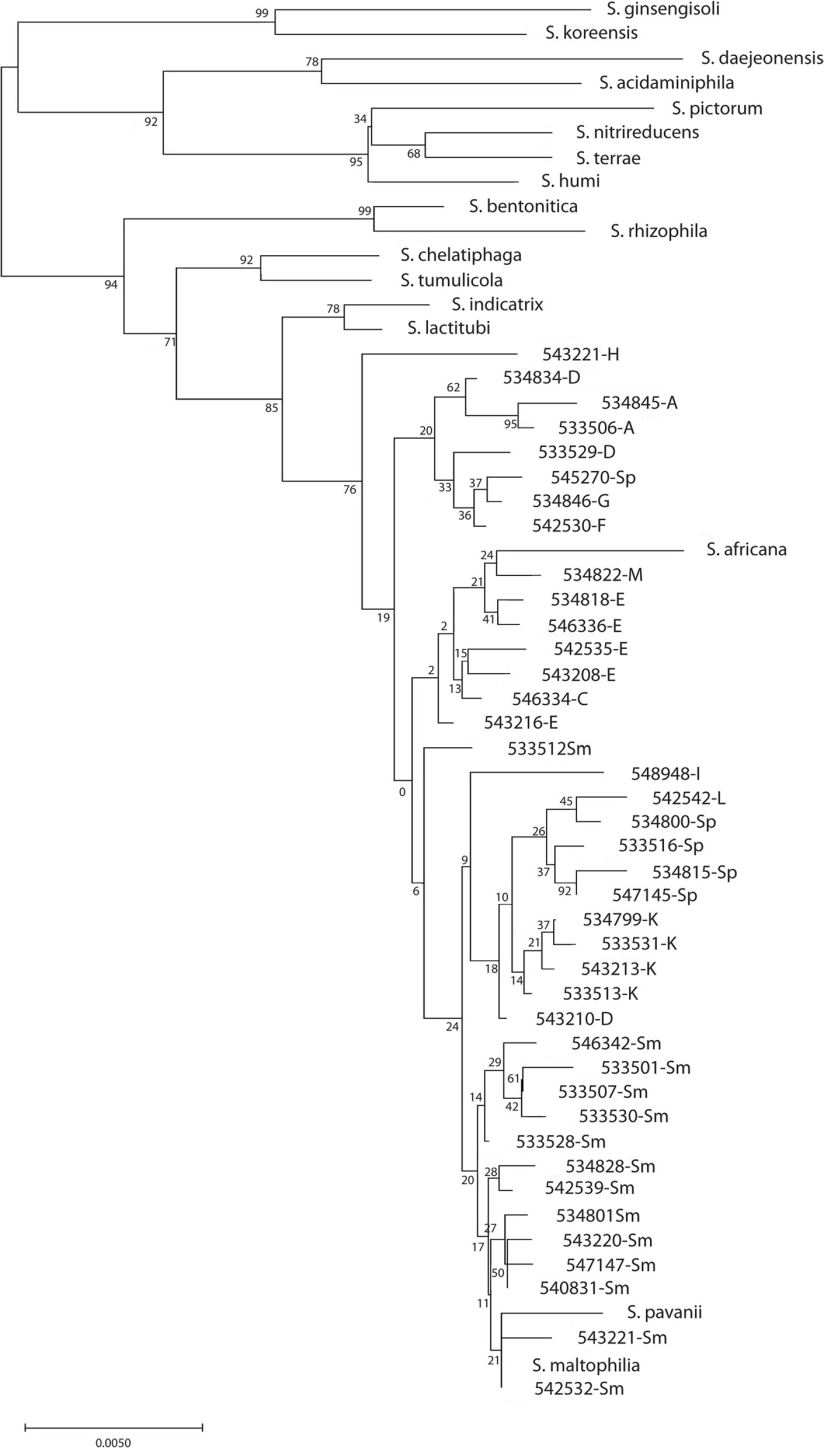
Evolutionary history of *Stenotrophomonas* strains belonging to unknown species based on concatenated alleles of the MLST scheme using 16S rRNA sequences was inferred using the Neighbor-Joining method. The optimal tree with the sum of branch length = 0.18050158 is shown. The percentage of replicate trees in which the associated taxa clustered together in the bootstrap test (1050 replicates) are shown next to the branches. The tree is drawn to scale, with branch lengths in the same units as those of the evolutionary distances used to infer the phylogenetic tree. The analysis involved 57 nucleotide sequences. There were a total of 1392 positions in the final dataset

A neighbor-joining tree of our isolates based on unique 16S rRNA sequences for each species combined with the 16S rDNA sequences of the type strains showed that the type strains clustered separately, with the exception of *S. maltophilia* and *S. pavanii*, which clustered together. Sequences of all isolates identified as *S. maltophilia* by MALDI-TOF/MS clustered together. However, they generally clustered by (putative) species with a number of exceptions, e.g., *S. maltophilia* isolate 533,512 and *S. pavanii* isolate 545,270 and the type strain. *S. africana,* which is not recognized as a separate species anymore but considered as synonymous with *S. maltophilia*, also clustered with the isolates that were initially identified as *S. maltophilia* (Fig. 3).

### Antibiotic resistance

**Fig. 4:**
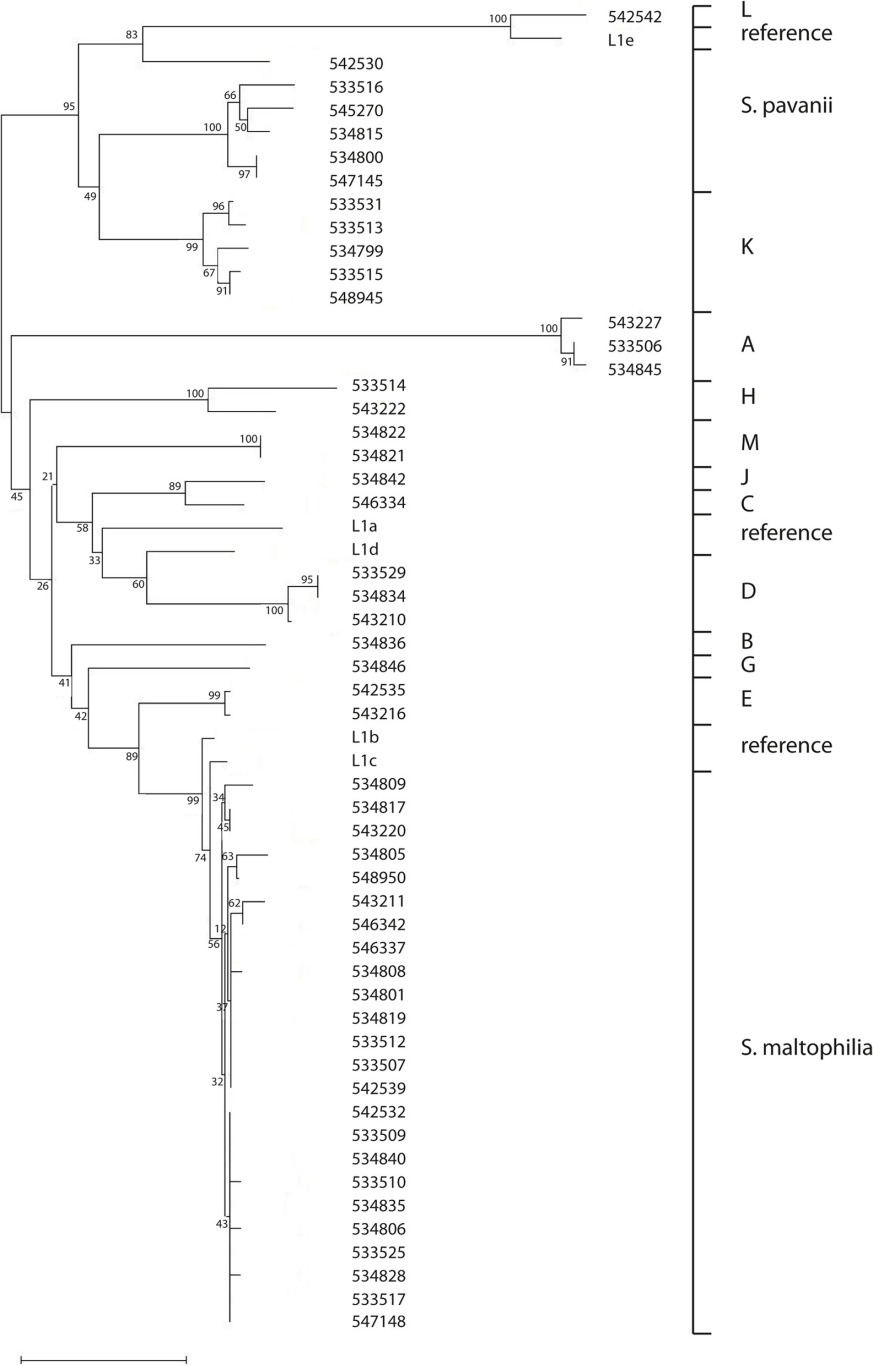
The evolutionary history of L1 ß-lactamases was inferred using the Neighbor-Joining method. One amino acid sequence per ST was included. L1a-L1d were used as reference sequences from literature (Walsh et al., 1994; Crowder et al., 1998). The optimal tree with the sum of branch length = 1.25626229 is shown. The percentage of replicate trees in which the associated taxa clustered together in the bootstrap test (1050 replicates) are shown next to the branches. The tree is drawn to scale, with branch lengths in the same units as those of the evolutionary distances used to infer the phylogenetic tree. The analysis involved 57 amino acid sequences. There were a total of 295 positions in the final dataset. No L1 sequences were found for the ST34, STH, and STI isolates. The letter behind the strain number indicate the presumptive species (Sm = *Stenotrophomonas maltophilia*, Sp = *S. pavani*) or putative novel species (A-M) as based on the analysis of the ANIb

**Fig. 5:**
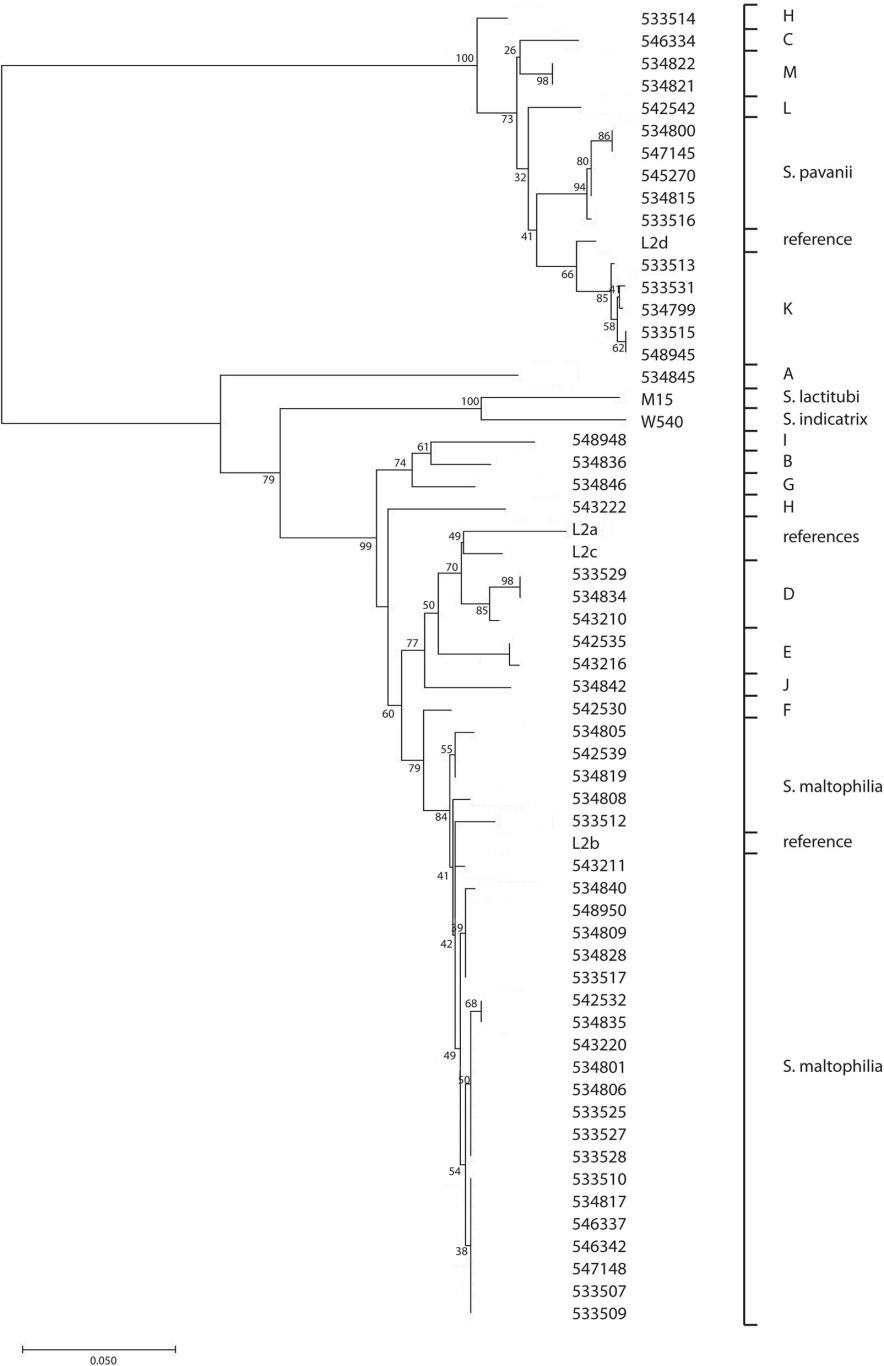
The evolutionary history of L2 ß-lactamases was inferred using the Neighbor-Joining method. One amino acid sequence per ST was included. L2a-L2d were used as reference sequences from literature (Walsh et al., 1997). Strains M15 and W540 are the type strains for *S. lactitubi* and *S. indicatrix*, respectively. The optimal tree with the sum of branch length = 1.04210294 is shown. The percentage of replicate trees in which the associated taxa clustered together in the bootstrap test (1050 replicates) are shown next to the branches. The tree is drawn to scale, with branch lengths in the same units as those of the evolutionary distances used to infer the phylogenetic tree. The analysis involved 59 amino acid sequences. There were a total of 305 positions in the final dataset. There were a total of 304 positions in the final dataset. No L2 ß-lactamase sequences for ST89 and ST215 isolates were found

In *S. maltophilia* the L1 and L2 ß-lactamases have been described [6–8]. Most isolates in our study encoded a L1 family ß-lactamase, but in four the gene could not be detected: 3 *S. maltophilia* isolates and the isolate of the putative species I. L2 was present in all isolates collected for this study, although only partial sequences were available for four isolates (two incomplete sequences and two truncated sequences with 24-132x sequence coverage). The type strains for *S. maltophilia* and *S. pavanii* contained the genes for both ß-lactamases. *S. indicatrix* and *S. lactitubi* harbored only the L2 gene, but the type strains of the other species lacked the genes for the L1 and L2 ß-lactamase. A Neighbor-Joining tree of the amino acid sequences of both ß-lactamases showed that these cluster according to the putative species A-M, *S. maltophilia*, and *S. pavanii* as defined by ANIb (Figs. 4 and 5). The two isolates from putative species H were an exception. These showed clear differences between the ß-lactamases, in particular for the L2 ß-lactamases.

All *S. pavanii* isolates contained an *aac (6′)-Iak* aminoglycoside resistance gene and all isolates that were *S. maltophilia* based on the ANIb had an *aph (3′)-IIc* aminoglycoside resistance gene. Based on other genes present on the same contig, these resistance genes were located chromosomally, which is consistent with their presence in all isolates (Table 1). Many *S. maltophilia* isolates (72%) also contained an *aac (6′)-Iz* aminoglycoside resistance gene, which appeared to be present on the chromosome. The data suggest that the presence or absence of *aac (6′)-Iz* is ST-dependent (Table 1). The putative species M encoded an *aac (6′)-Ib3* and *ant (2″)-Ia* aminoglycoside resistance gene; both genes appeared to be chromosomally located. The other putative species lacked aminoglycoside resistance genes (Table 1); these generally displayed lower tobramycin MICs, although some were also > 128 mg/L. Seven *S. maltophilia* isolates (11%) also had acquired aminoglycoside resistance genes. Although the majority of the isolates lacked acquired aminoglycoside resistance genes, isolate 534,804 encoded 7 of these genes (Table 1). This isolate also harbored *cmx*, *tet(G)*, and *sul1*, encoding resistance against phenicols, tetracyclines, and sulfonamides respectively. Resistance genes other than those encoding aminoglycoside resistance were observed in isolate 534,806. These were *dfrA15*, *tet(A)*, and *sul1* encoding trimethoprim, tetracycline and sulfonamide resistance (Table 1). The *sul1* gene was associated with a class 1 integron, but the integrase encoding genes could not be identified.

Fluoroquinolone resistance is associated with over expression of the efflux pumps SmQnr, SmeDEF or SmeVWX. For SmeVWX a number of changes in the regulator SmeRv are associated with overexpression [13–16], and the following mutations with amino acid changes were detected in our isolates: G266D in isolates 553,507 and 543,203; G266S in 533,509; C310Y in 533,517 and 533,533; and C310W in 534,828. The MICs for ciprofloxacin ranged from 2- > 32 μg/mL. Isolates 533,532, 534,846, 545,270, and 547,144 were truncated at positions 305, 305, 207, and 173, respectively; the range of ciprofloxacin MICs in these isolates was 2–16 μg/mL.

The only susceptibility breakpoint that EUCAST has currently defined for *S. maltophilia* is to co-trimoxazole; strains with a MIC ≤4 mg/L are susceptible with increased exposure (i.e. using a dose of 1440 mg q12h) [17]. Twenty-five of the 65 *S. maltophilia* isolates were resistant (39%). Applying the *S. maltophilia* co-trimoxazole breakpoint to *S. pavanii*, two of six isolates would be resistant (Supplementary Table 1). Based on this breakpoint, 14 of the 40 isolates (35%) with undefined taxonomic status would be resistant to co-trimoxazole.

### Putative virulence genes and expression of protease and esterase activity

The genes for the proteases StmPr1, StmPr2, and StmP3, as well as the DNase, phospholipase C and D, esterase, fimbriae, TadE-like protein, the RpfC regulator, glucose-1-phosphate thymylyltransferase, phosphoglucomutase/phosphomannose bifunctional protein, and Ax21 outer membrane protein were present in nearly all isolates, independent of their taxonomic status; only six isolates lacked one or a few of these genes (Table 2, Supplementary Table 2). The genes for the type IV pilus machinery, Xps type II secretion system, and hemolysin were only found in *S. maltophilia* and in two isolates in the group of putative species (B and J) (Table 2, Supplementary Table 2). The genes for the other virulence factors showed a more mixed picture (Table 2, Supplementary Table 2), although generally the virulence factors were more frequently present in the *S. maltophilia* than in the putative novel species.

**Table 2:** Percentage isolates positive in each species group

Function	Genetic identifier	Total (n/%)	***S. maltophilia*** (n/%)	Others (n/%)
alkaline serum protease stmPr1	*stmPr1*	111/100.0	65/100.0	46/100.0
protease	*stmPr2*	111/100.0	65/100.0	46/100.0
protease	*stmPr3*	110/99.1	64/98.5	46/100.0
Xps type II secretion system	*smlt0687–0697*	62/55.8	60/92.3	2/4.3
DNase	*smlt3212*	111/100.0	65/100.0	46/100.0
phospholipase C	*smlt1755*	110/99.1	65/100.0	45/97.8
phospholipase D	*smlt3521*	110/99.1	65/100.0	45/97.8
esterase	*smlt3773*	111/100.0	65/100.0	46/100.0
polysaccharide lyase	*smlt1473*	85/76.6	55/84.6	30/65.2
nitrate reductase	*narG*	53/47.7	41/63.1	12/26.1
fimbriae	*smf-1*	109/98.2	64/98.5	45/97.8
TadE-like protein	*smlt2869 achter*	108/97.3	64/98.5	44/8.7
giant cable pilus-like protein	*smlt3830voor*	93/83.8	64/98.5	29/63.0
afimbrial adhesin	*smlt4423voor*	56/50.5	39/60.0	17/37.0
type IV pilus machinery	*smlt1621–1627*	61/55.0	59/90.8	2/4.3
ankyrin repeat domain-containing protein	*smlt3054*	27/24.3	16/24.6	11/23.9
filamentous hemagglutinin	*fha*	61/55.0	55/84.6	6/13.0
hemolysin	*hly*	14/12.6	14/21.5	0/0.0
regulator RpfC	*rpfC*	111/100.0	65/100.0	46/100.0
regulator RpfD	*rpfF*	84/75.7	56/86.2	28/60.9
glucose-1-phosphate thymdylyltransferase	*rmlA*	110/99.1	65/100.0	45/97.8
phosphoglucomutase/phosphomannose bifunctional protein	*spgM*	110/99.1	65/100.0	45/97.8
Ax21 outer membrane protein	*ax1*	110/99.1	65/100.0	45/97.8

Extensive sequence variation was observed for the type IV pilin adhesion precursor gene (Supplementary Table 2). Seven isolates exhibited only partial genes for filamentous hemagglutinin. DNase also showed some variation. Two isolates (545,270 and 533,529) had a truncated esterase. The glucose-1-phosphate thymylyltransferase was present in all strains and some variation was observed. Variation occurred also within STs (ST5, ST15, ST39, ST162). The Ax21 outer membrane protein was nearly identical within *S. maltophilia* and *S. pavanii* with a variation in only one amino acid position, but it was more variable within the isolates of putative species B-M (variations in 31 positions). Ax21, DNase, phospholipase D sequences in *S. maltophilia* cluster together as do the *S. pavanii* sequences. The sequences of the putative species form several clusters (data not shown).

The RPF (Regulation of Pathogenicity Factors) cluster of genes encodes a two-component system with a sensor and phosphorylation protein, as well as enoyl-CoA hydratase and a long chain-fatty-acid-CoA-ligase; together this composes a quorum sensing system with the fatty acid cis-11-methyl-2-dodecenoic acid (DSF) as the signal [18, 19]. The RPF cluster showed considerable sequence variation, especially in the sensor protein, with four main groups. (Supplementary Fig. 2).

The phenotypic expression of protease and esterase activity was also assessed: only 71.6% of the isolates exhibited protease activity and 40.4% exhibited esterase activity. Both the protease and the esterase activities showed associations with (putative) species or STs.

## Discussion

The taxonomy of the genus *Stenotrophomonas* is complex and requires revision [4]. The individual *Stenotrophomonas* species recognized by the List of Prokaryotic names with Standing in Nomenclature may be distinguished from each other by using the conservative cut-off for species of 0.95 in the ANIb (Fig. 1, Supplementary Fig. 1) [1, 20]. Using this same criterion, 65 of 111 isolates collected for this study and identified by MALDI-TOF/MS as *S. maltophilia* would belong to this species. Six isolates were *S. pavanii*; the other isolates would represent a total of 13 different novel species, which we here indicate as putative species A-M. Isolates that cluster as putative species B-M cluster more closely to *S. maltophilia* than to the type strains of other *Stenotrophomonas* species, suggesting that either the divergence of these putative species is more recent or that genetic exchange between these isolates has been more common.

A neighbor-joining tree of the concatenated alleles of the *S. maltophila* MLST scheme used to compare the ANIb results with those of Ochoa-Sánchez and Vinuesa showed that most groups of isolates or new species grouped comparably (Fig. 2) [4]. Group #1 isolates clustered with the *S. pavanii* type strain, and also our pairwise clustering suggests that these isolates are *S. pavanii*. The previously identified group #6 clustered with the *S. maltophilia* type strain as well as with a number of our isolates. Group #9 was previously proposed as a novel species (Smc4), but, based on their clustering with the type strain in the MLST based phylogeny, the isolates in these group are most likely either *S. indicatrix* or *S. lactitubi*. Several previously proposed lineages clustered with the putative species in the ANIb analysis, e.g., lineage #3, #4, #5, #7 with putative species L, E, J, and B, respectively. Some of the putative species from the ANIb clustered with isolates that previously belonged to a different species now considered synonymous with *S. maltophilia*, such as *P. beteli*. Our data suggest that these isolates differ significantly from *S. maltophilia* and may indeed represent different species.

Some groups ((D), (F), Smc1, and Smc2) did not group in our analysis. This may be due to a different algorithm to generate the trees, but another explanation is that concatenated STs are not optimal for defining species [3, 4]. However, it should be noted that the bootstrap values for some branches are low, indicating low confidence for the branching.

To further elucidate the taxonomic position of our isolates, a 16S rDNA sequence analysis was performed. This analysis was basically in agreement with the ANIb analysis. Remarkably, the 16S rDNA sequence of the *S. pavanii* type strain clustered closely with that of the *S. maltophilia* type strain and with the *S. maltophilia* isolates collected for this study, whereas our other *S. pavanii* isolates clustered elsewhere (Fig. 3). As already suggested by the ANIb, the 16S rDNA data suggest that *S. maltophilia*, *S. pavanii* and the putative new species are still closely related. Based on 16S analysis, *S. africana*, which is currently considered synonymous with *S. maltophilia*, should be considered as a separate species.

All our isolates harbored a chromosomally encoded L1 and L2 ß-lactamase. However, the (type) strains of species other than *S. maltophilia* and *S. pavanii* lacked these ß-lactamases, except for *S. indicatrix* and *S. lactitubi*, which encoded the L2 gene. A Neighbor-joining analysis of the amino acid sequences was in agreement with the results of the ANIb and concatenated STs (Fig. 4, Fig. 5): isolates identified as *S. maltophilia* in the ANIb clustered together with limited sequence variability; the other (putative) species displayed more diversity. The L1 ß-lactamase hydrolyzes carbapenems and other ß-lactam antibiotics (but not monobactams), whereas L2 ß-lactamase is a serine hydrolase that acts as a cephalosporinase. *Stenotrophomonas* MICs for imipenem and meropenem are generally high; more variation is seen for ceftazidime. The considerable sequence variation for both ß-lactamases, which was already described when the first genes were sequenced, may at least in part explain differences between isolates [6–9].

All *S. pavanii* isolates contained a chromosomally encoded *aac (6′)-Iak* aminoglycoside resistance gene and all isolates that were *S. maltophilia* based on the ANIb had an *aph (3′)-IIc* aminoglycoside resistance gene, whereas the other isolates lacked chromosomally encoded aminoglycoside resistance genes. *S. maltophilia* and *S. pavanii* tended to have higher MICs for tobramycin than the putative species (Supplementary Table 1). However, this correlation was not perfect, suggesting that other factors play a role in expression and thereby resistance, including regulation of aminoglycoside resistance genes. In addition to *aph (3′)-IIc* some *S. maltophilia* had *aac (6′)-Iz*, a chromosomally encoded aminoglycoside resistance gene (Table 1).

Only a few isolates possessed acquired resistance genes (Table 1), but one isolate encoded 7 different ones, including a *sul1* resistance gene. *Sul1* is associated with class 1 integrons [21], but presence of an integron could not be confirmed. Nevertheless, this suggests that *S. maltophilia* can acquire plasmids or transposons with class 1 integrons, as has been previously described [21, 22]. Differences between the (presence of) chromosomally encoded antibiotic resistance genes further supports the hypothesis that our isolates included different species (*S. maltophilia*, *S. pavanii* and a set of putative novel species). Although the chromosomally encoded ß-lactamases and aminoglycoside resistance genes may be clinically relevant, their function is probably to aid in competition with other micro-organisms in their natural niche.

The extensive resistance pattern of *S. maltophilia* severely limits the antibiotic treatment options for infections, and only co-trimoxazole is considered a reliable treatment option. Described alternatives include fluoroquinolones and tetracyclines [10], but the MICs may vary considerably. Currently little is known about the genetic factors determining resistance to these antibiotics with the exception of fluoroquinolone resistance, which is associated with overexpression of efflux pumps. These are either SmQnr, SmeDEF or SmeVWX [14–16]. Overexpression of SmeVWX is associated with amino acid changes in its repressor, SmeRv [15, 16]. Five isolates had previously described amino acid substitutions; however, their ciprofloxacin MICs ranged from 2 to > 32 mg/L. For the newly described variants (a C310W in 534,828 and truncated sequences) the range was 2 to 16 mg/L. No mutations in the quinolone resistance determining regions of *gyrAB* and *parCE* were observed [13].

The presence of the genes for the proteases StmPr1, StmPr2, and StmP3, as well as the DNase, phospholipase C and D, esterase, fimbriae, TadE-like protein, and the RpfC regulator genes in all or nearly all isolates (Table 2, Supplementary Table 2). However, somewhat lower percentages have been reported in literature. StmPr1, StmPr2, esterase, and fimbriae were present in more than 90% of the isolates in a collection from CF patients attending a pediatric hospital in Rome, Italy [23]. Possibly, the collections differed in the distribution of different (putative) species, and that these genes do not belong to the core genome of all (putative) species.

Differences in the type IV pilus adhesion precursor should likely be sought in the different adherence properties that may lead to advantages in different natural niches.

StmPr1, − 2, and − 3 degrade a wide variety of extracellular matrix components. StmPr1 degrades collagen type I and IL-8, and can kill A549 lung epithelial cells; StmP3 degrades fibronectin, fibrinogen, and IL-8, and contributes to cell rounding and detachment in vitro; the activities of StmPr2 have not exactly been defined, but this protease contributes to degradation of extracellular matrix proteins and cell rounding. The three proteases are secreted in Xps-dependent manner [24, 25]. However, the genes encoding Xps type II secretion system could only be identified in 90% of the *S. maltophilia* isolates and in putative species B and J andabsent in the other (putative) species. Since phenotypic protease activity did not correlate with species or STs, proteases may likely also be secreted by an alternative mechanism. All isolates contained protease genes and an esterase gene, but phenotypic expression of protease and esterase activity was found in only 71.6 and 40.4% of the isolates respectively. This suggests that the regulation of expression of these activities is complex. Alternatively, additional proteases may be present which are secreted by a different mechanism.

Polysaccharide lyase degrades alginate, poly-ß-D-glucuronic acid and hyaluronic acid [26]. These first two compounds are found widely in nature and hyaluronic acid is an important constituent of human skin, but it is also found elsewhere in the human body. The polysaccharide lyase was present in more than three quarters of the isolates, and its absence/presence did not follow the (putative) species boundaries. The absence of the gene in many isolates obtained from CF and lung infections indicate that it is not essential for colonization or infection of the human lung.

The secreted ankyrin-repeat protein, which interacts with actin, was present in only approximately a quarter of the isolates [27]. This interaction is thought to alter the cytoskeletal structure. However, StmPr1 and StmPr2 also contribute to this process [25].

The RPF quorum sensing system showed considerable sequence variability, in particular for the sensor protein (Supplementary Fig. 2). This quorum sensing system has been reported to regulate motility, biofilm development, antibiotic resistance and virulence in *S. maltophilia*, but the implications for (CF) lung infections are not immediately clear [18, 19, 28]. It has been reported that the signal molecule DSF generated by the expression of the RPF system influences *Pseudomonas aeruginosa* biofilm formation and antibiotic resistance [29–31]. Some isolates lack a 190 amino acid sequence in the sensor domain region in the middle of the protein. Huedo et al showed sequence variation in the RPF cluster of *S. maltophilia* and designated two variants: *rpf-1* and *rpf-2* [32]. The first is more similar to the system found in *Xanthomonas*, whereas the latter is more similar to the system in *Pseudoxanthomonas*, *Arenimonas*, and *Lysobacter*. In a *rpf-2* system, with a shorter sensor domain, it appears that some quorum sensing signal DSF needs to be present for activation of DSF synthesis. It has been speculated that under some conditions the *rpf-2* system saves energy, and that its activation is dependent on the presence of other bacterial isolates or species [32].

The majority of *S. maltophilia* isolates are capable of biofilm production, but marked differences have been observed. On average more biofilm formation on polystyrene was observed by non-CF isolates than by CF isolates, which in turn displayed more biofilm formation than environmental isolates, but large variation among isolates within the groups was present [33]. Variable biofilm formation was also observed on IB3–1 bronchial cells in vitro [34].

The *spgM*, and *rmlA* genes, encoding aphosphoglucomutase/phosphomannose bifunctional protein and glucose-1-phosphate thymylyltransferase, respectively, have been implicated in biofilm formation and were reported in 83.3 and 87.5% of 37 isolates tested [35]. However, all our isolates contained these genes; therefore, either there were differences in the populations tested, or the PCR used in prior studies did not detect all genes due to sequence variation in the primer region(s). Although the RPF system has been reported to influence biofilm formation, no correlation with the detection of *rpfF* by PCR was found [35]. The nitrate reductase has also been associated with biofilm by enabling growth in micro-oxic conditions [36]. Nearly half of our isolates harbored the *narG* gene encoding the nitrate reductase; this was in agreement with a previous study that reported 37/63 positive isolates [36]. The Ax21 outer membrane protein is also involved in biofilm formation, as well as motility, reduced tolerance to tobramycin, and virulence in an insect model, but its regulation and mode action of action have not been resolved [37].

Although these data help to identify putative virulence their role should ultimately be proven in vivo.

## Conclusions

This study has shown that either *S. maltophilia* is highly heterogeneous and *S. pavanii* should be included in this species, or that *S. maltophilia* is more limited and many of our isolates belong to currently undescribed and unnamed species. The putative novel *Stenotrophomonas* species recovered from patient samples and identified by MALDI-TOF/MS as *S. maltophilia*, differed from *S. maltophilia* in resistance and virulence genes, and, as a consequence, possibly in pathogenicity. Revision of the *Stenotrophomonas* taxonomy is needed to reliably identify strains within the genus and elucidate the role of the different species in disease.

## Methods

### The study

The strains were collected for the iABC-project, in which novel antibiotics for CF and bronchiectasis are being developed. A total of 111 isolates were included in the study. These isolates were recovered from respiratory samples of CF (*n* = 103) and diverse pulmonary infections (*n* = 8) between 2003 and 2016 from five different countries: Australia (*n* = 1), United Kingdom (*n* = 41), Spain (*n* = 35), the Netherlands (*n* = 33), and USA (*n* = 1) (Table 1). Matrix-assisted laser desorption/ionization time of flight mass spectrometry (MALDI-TOF/MS, Bruker) identified all isolates as *S. maltophilia*.

The aim of the present study was to determine the taxonomic position of *S. maltophilia* isolates from persons with CF and patients with other chronic respiratory infections, and to characterize their antibiotic resistance genes and virulence factors.

### Whole genome sequencing

Bacterial DNA was purified using the Qiacube with the DNeasy Blood & Tissue kit with the enzymatic lysis protocol (Qiagen, Carlsbad, CA) and used to prepare a library for sequencing with the MiSeq or Nextseq (Illumina, San Diego, CA) platforms, using the Nextera XT library prep kit (Illumina). Contigs were assembled with SPAdes genome assembler v.3.6.2. The assembled contigs were analyzed for the presence of resistance genes by ResFinder [38, 39] from the Center for Genomic Epidemiology (DTU, Copenhagen, Denmark) [39]. Multi-locus Sequence Typing (MLST) was performed using PubMLST using the scheme for *S. maltophilia* [40]. Novel alleles and sequence types (ST) were submitted to the database.

### Analysis of average nucleotide identity by BLAST (ANIb)

Average nucleotide identity (ANI) among the genomes was calculated using ANIb algorithm of pyani tool version 0.2.3 [41], which uses nucleotide BLAST (version 2.2.28+) alignment for whole genome alignment. Genomes were fragmented into genomic fragments of 1020 bases long. After pairwise alignments of all fragments of each genome, ANI was calculated as the percentage of nucleotide identity for matching regions of all genomes. In biclustering analysis of ANI scores, complete linkage was used as a hierarchical clustering method with the Euclidean distance metric. A heatmap of all genomes was generated using biclustering, where a color scale bar shows the pairwise ANI scores above 75% are shown in a color range starting from blue (ANI 75%) through white to red (ANI 100%). In the heatmap, each species is shown in a different color next to a leaf node of a tree. A heatmap of all genomes was generated using biclustering, with a color scale bar showing the pairwise ANI score for values above the threshold value of 0.75. A cut-off of 0.95 was used to define groups, which is also the conservative cut-off used to define species [20, 42].

### Phylogenetic trees

Neighbor-joining trees of the concatenated alleles of the MLST scheme for *S. malthophilia*, the 16S rRNA gene, and the L1 and L2 ß-lactamase sequences were generated with WebPrank and Clustal Omega for the alignments, and with MEGA X for the generation of the tree, using 500 bootstraps [40, 43]. The trees were drawn to scale, with branch lengths in the same units as those of the evolutionary distances used to infer the phylogenetic tree. The evolutionary distances were computed using the Maximum Composite Likelihood method, in the units of the number of base substitutions per site for the 16S rDNA sequences. For the other sequences the evolutionary distances were computed using the Poisson correction method, in the units of the number of amino acid substitutions per site [44–48]. For the ß-lactamase sequences only one sequence per ST was included. KODON (Applied Maths, Belgium) was used for the whole genome alignments.

The whole genome sequences of the type strains of all species except *S. tumilicola* were available for analysis.

### Antimicrobial susceptibility testing

Minimum inhibitory concentrations (MICs) were determined by the standard ISO broth microdilution method with frozen panels (Trek Diagnostic Systems, Westlake, OH) using EUCAST methodology [17]. The antimicrobial agents and the concentration ranges tested were as follows: aztreonam (0.25–256 mg/L); ceftazidime (0.25–256 mg/L); ciprofloxacin (0.03–32 mg/L); colistin (0.25–16 mg/L); tobramycin (0.125–128 mg/L); imipenem (0.125–128 mg/L); meropenem (0.06–64 mg/L); and trimethoprim/sulfamethoxazole (co-trimoxazole) (0.06–32 mg/L). The susceptibility data for the isolates recovered from CF patients were previously reported [49].

### Protease and esterase activity

Bacteria were grown to 1 OD_600_ in LB broth and 2 μl was spotted onto agar with 2% skim milk and onto plates with tributyrin-Arabic gum, and incubated overnight at 37 °C to respectively assess protease activity, and esterase activity [50, 51].

## Supplementary Information

**Additional file 1.**

**Additional file 2.**

**Additional file 3.**

**Additional file 4.**

## Data Availability

The sequence data have been submitted to GenBank (BioProject ID: PRJNA718312 available at https://dataview.ncbi.nlm.nih.gov/object/PRJNA718312?reviewer=epjohsfos888drv6cjnimg3f8s). Data have also been submitted to the DRYAD database. These data have a temporary link: https://datadryad.org/stash/share/keoI3aRF2r55sXSotR_oxlA6yyfdd1KMfsxG-N_-_IQ.

## Abbreviations

ANIb: Average nucleotide identity by BLAST
CF: Cystic fibrosis
MALDI-TOF/MS: Matrix-assisted laser desorption/ionization time of flight mass spectrometry
MB: Megabase
MIC: Minimum inhibitory concentrations
MLST: Multi-Locus Sequence Typing
RPF: Regulation of Pathogenicity Factors
*S. acidaminiphila*: *Stenotrophomonas acidaminiphila*
*S. bentonitica*: *Stenotrophomonas bentonitica*
*S. chelatiphaga*: *Stenotrophomonas chelatiphaga*
*S. daejeonensis*: *Stenotrophomonas daejeonensis*
*S. ginsengisoli*: *Stenotrophomonas ginsengisoli*
*S. humi*: *Stenotrophomonas humi*
*S. indicatrix*: *Stenotrophomonas indicatrix*
*S. koreensis*: *Stenotrophomonas koreensis*
*S. lactitubi*: *Stenotrophomonas lactitubi*
*S. maltophilia*: *Stenotrophomonas maltophilia*
*S. nitrireducens*: *Stenotrophomonas nitrireducens*
*S. pavanii*: *Stenotrophomonas pavanii*
*S. pictorum*: *Stenotrophomonas pictorum*
*S. rhizophila*: *Stenotrophomonas rhizophila*
*S. terrae*: *Stenotrophomonas terrae*
*S. tumulicola*: *Stenotrophomonas tumulicola*
ST: Sequence type
