# Supplementary material for: Taxonomic position, antibiotic resistance and virulence factor production by Stenotrophomonas isolates from patients with cystic fibrosis and other chronic respiratory infections
Source: BMC Microbiol. 2022 May 12;22:129. doi: 10.1186/s12866-022-02466-5 (PMC9097388; doi:10.1186/s12866-022-02466-5)
Supplement: Supplementary file 1 — Additional file 1. [file 12866_2022_2466_MOESM1_ESM.pdf]

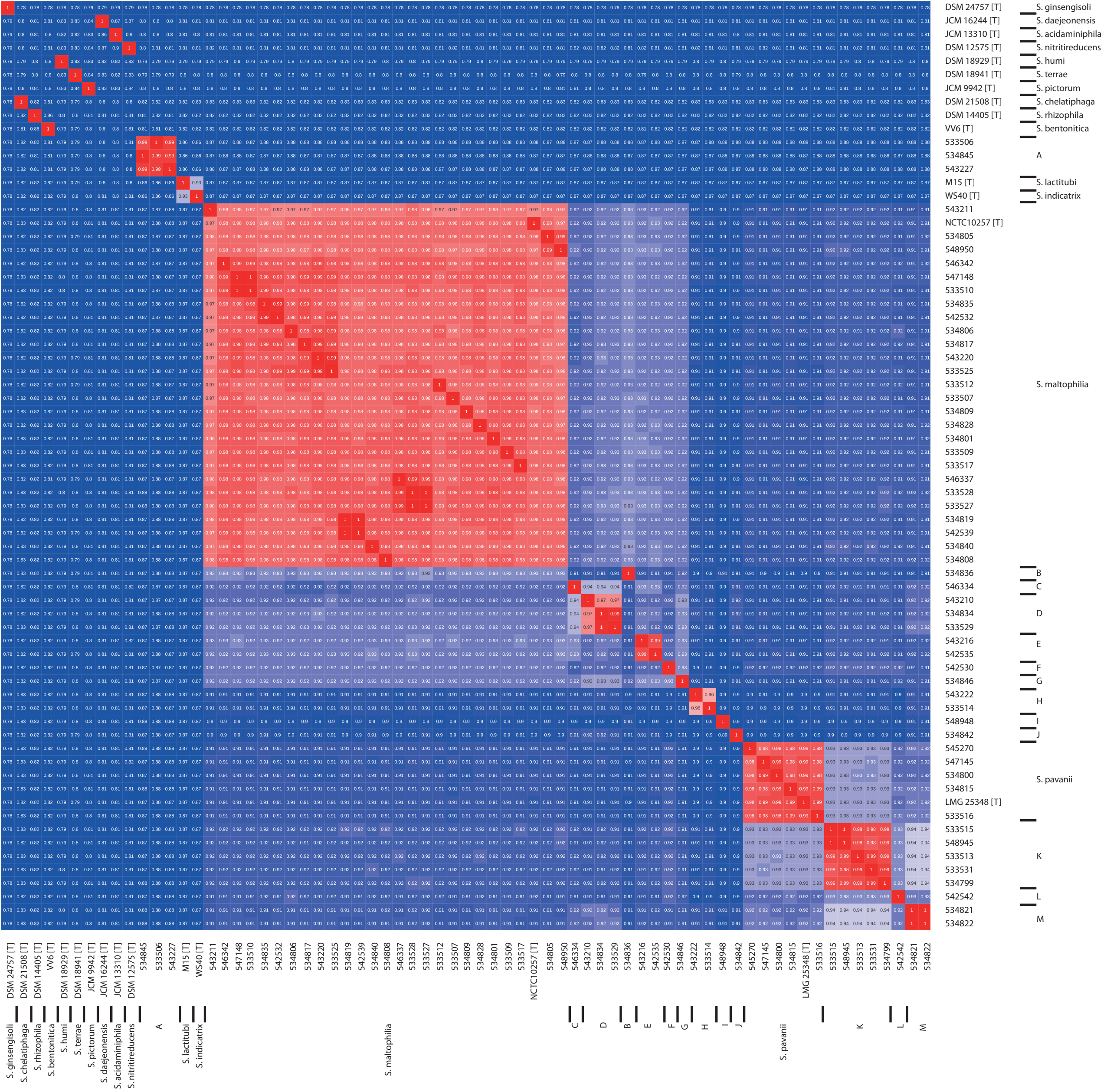


**Supplementary figure 1**

Heatmap based on percentage of ANIb for strains belonging to the different STs that were analyzed in this study (one isolate per ST was included) as well as available type strain sequences. The color bar shows the percentage of ANIb between any two strains starting from blue (0.8 or 80%) to red (1 or 100%); the blue/red cutoff is 0.94/0.95 (see Methods section for more details). Type strains are indicated by [T] behind the strain identification. The putative species (see text) are indicated at the right.
