# Supplementary material for: Taxonomic position, antibiotic resistance and virulence factor production by Stenotrophomonas isolates from patients with cystic fibrosis and other chronic respiratory infections
Source: BMC Microbiol. 2022 May 12;22:129. doi: 10.1186/s12866-022-02466-5 (PMC9097388; doi:10.1186/s12866-022-02466-5)
Supplement: Supplementary file 4 — Additional file 4. [file 12866_2022_2466_MOESM4_ESM.pdf]

```
543220      ---------------------------------------------------MQDLFARVR    9
543211      ---------------------------------------------------MQDLFARVR    9
543223      ------------------------------------MRRAFDGVRTPGAGRVKRLLSRLR    24
533516      MDHGSHPGLSLRPAWPAVRSPMRGRAAARPRTAGPDLRRAFDGVRAPGAGRVKRLLSQLR    60
                                                            :: *::::*

543220      LRLAQRPDSEHGQAIVRVVLISLILVYVLLSGARLH-RLDQYQGALATALTGLGLSLLLF    68
543211      RRLAQRPDSEHGQAIVRVVLISLILVYVLLSGARLH-RLDQYQGALATAMTGLGLSLLLF    68
543223      LRLSQRQDSEHGQQIVRIVLISLILAYVLLPAPRHDLPHGQYVGVLAIVLTGLSLSLLLF    84
533516      LRLSQRQDSEHGQQIVRIVLISLILAYVLLPAPRHDLPHTQYVGVLAIVLTGLSLSLLLF    120
            **:** ****** ***:*******.**** . * .    ** *.** .:***.******

543220      GWLLWKPQRSDLRRVAGMLADYGLIAIGMIQMGEPLAWVYAVVMWVTVGNGLRFGPRYLT    128
543211      GWLLWKPQRSDLRRVAGMLADYGLIAIGMIQMGEPLAWVYAVVIWVTVGNGLRFGPRYLT    128
543223      GWLLWRPERSDARRVLGMLADYGLIAAGMVQMGEPLAWVYIVVMWVTVGNGMRFGNHYLY    144
533516      GWLLWRPARSDPRRVLGMLADYGLIAAGMVQMGEPLAWVYIVVMWVTVGNGMRFGNHYLY    180
            *****:* *** *** ********** **:********** **:*******:*** :**

543220      AAIVLAMISFGTTVLLTPYWQANLGLAISLWLGLLAVPLCCSSLLRRRMRHPTMDTIPAA    188
543211      AAIVLAMISFGTTVLLTPYWQANLGLAISLWLGLLAVPLCCSSLLRRRMRHPTMDTIPAA    188
543223      VAVAMALVSFGFTVLQTPYWMQNFRLAIGLWLGLAAVPL---------------------    183
533516      VAVAMAMVSFGCTVLLTPYWQQNFRLAIGLWLGLAAVPL---------------------    219
            .*:.:*::*** *** ****  *: ***.***** ****

543220      GSDKRSLLARFKQRLSGREDSEHAQNLIRIIITALFISYLGWRSLSGGGEALTATWLILA    248
543211      GSDKRSLLARFKQRLSGREDSEHAQNLIRIIITALFISYLGWRSLSGGGEALTATWLILA    248
543223      ------------------------------------------------------------    183
533516      ------------------------------------------------------------    219


543220      FELTISAGLLTAILVNPGVSHVRRVIGMLTDYTCMAWIMTIQGEPAAPLYAVMLWVTIGN    308
543211      FELTISAGLLTAILVNPGVSHVRRVIGMLTDYTCMAWIMTIQGEPAAPLYAVMLWVTIGN    308
543223      ------------------------------------------------------------    183
533516      ------------------------------------------------------------    219


543220      GMRYGSTYLRVATAMGCLAFLCTVMLSPYWRGHDYLGWGLLLGLGAIPLYFDSLLHALTR    368
543211      GMRYGSTYLRVATAMGCLAFLCTVMLSPYWRGHDYLGWGLLLGLGAIPLYFDSLLHALTR    368
543223      -------------------------------------------------YFSTLLRQLTE    194
533516      -------------------------------------------------YFSTLLRQLTE    230
                                                             **.:**: **.

543220      AVAEARQANEAKSRFLANMSHEFRTPLNGLAGMTEVLATTRLDDEQRECLNTIQASTRSL    428
543211      AVAEARQANEAKSRFLANMSHEFRTPLNGLAGMTEVLATTRLDDEQRECLNTIQASTRSL    428
543223      AMAEARRASEAKSRFLANMSHEFRTPLNGLNGMTEVLATTRLDDEQRECLSTIQASSRSL    254
533516      AMAEARRASEAKSRFLANMSHEFRTPLNGLNGMTEVLATTRLDDEQRECLNTIQASSRSL    290
            *:****:*.********************* *******************.*****:***

543220      LALVEEVLDISAIEAGKVRVEWHEFALADVLQAINLILTPQTRSKSLDYRVQVDDNVPPR    488
543211      LALVEEVLDISAIEAGKVRVEWHEFALADVLQAINLILTPQTRSKSLDYRVQVDDNVPPR    488
543223      LALVEEVLDISAIEAGKLRVVAEDFAVTDVIQAIGLILMPQARAKGLDYRVKVADGVPPR    314
533516      LALVEEVLDISAIEAGKLRVVAEDFAVADVIQAIGLILLPQAKAKRLDYRVKVADGVPPR    350
            *****************:**  .:**::**:***.*** **:::* *****:* *.****

543220      LLGDAGHLQQILLNLMGNAVKFTDSGYVHLRVSSPDALGSERMRLRFEIVDTGIGVPVAL    548
543211      LLGDAGHLQQILLNLMGNAVKFTDSGYVHLRVSSPDALGSERMRLRFEIVDTGIGVPVAL    548
543223      VRGDVGHLRQILLNLAGNAVKFTDHGRVEIRVGVVHADTSGAVRLRFDIYDTGIGVAPAM    374
533516      VRGDVGHLRQILLNLAGNAVKFTDHGRVEIRVGVVQADASGAVRLRFDILDTGIGVPPAM    410
            : **.***:****** ******** * *.:**.  .*  *  :****:* ******  *:

543220      RGRLFNAFEQGDVGLARRHEGTGLGTAIAKGLVESMGGQVGYAPNSPRGSVFWVEITLEV    608
543211      RGRLFNAFEQGDVGLARRHEGTGLGTAIAKGLVESMGGQVGYAPNSPRGSVFWVEITLDV    608
543223      RARLFDAFEQADVSMARRHEGTGLGTTIAKGLVEAMDGDIGYLENPPRGSHFWVELPFAP    434
533516      RARLFDAFEQADVSMARRHEGTGLGTTIAKGLVEAMDGDIGYLENPPRGSHFWVELPFAP    470
            *.***:****.**.:***********:*******:*.*::**  * **** ****: :

543220      ADTSSAPAVPS-----EVAEAGNVIAFSNPFLRHRARVRSLRVLVADDHEANRMVLQRLL    663
543211      ADTSSAPAVPS-----EVAEAGNVIAFSNPFLRHRARVRSLRVLVADDHEANRMVLQRLL    663
```

```
543223      PQPMVPGAVPTLSGEEDAGPGGNVIAFADPFLRHRARVRSMQILVADDHEANRMVLQRLL    494
533516      PQPQVPGAVPSLSGDEGAGPGGNVIAFADPFLRHRARVRSMQILVADDHEANRMVLQRLL    530
             :     ***:      .. .******::***********:::*****************

543220      EKAGHKVTCVNGGAEVLDALEQASYDAAIVDLHMPGMSGLDLLKQLRVMQASGMPYVPVM    723
543211      EKAGHKVTCVNGGAEVLDALEQASYDAAIVDLHMPGMSGLDLLKQLRVMQASGMPYVPVM    723
543223      QKAGHRVLCVDGGEAVLDALADSEFDAVIVDLHMPGMSGLDMLKELRVMQAGGGPRTPVL    554
533516      QKAGHRVLCVDGGEAVLDALADSEFDAVIVDLHMPGMSGLDMLKELRVMQAGGGPRTPVL    590
            :****:* **:**  ***** ::.:**.*************:**:******.* * .**:

543220      VLSADVTPESILRCEQAGARAFLAKPVVATRLLEVLADVAANTRAEP--GIQSVPALPVG    781
543211      VLSADVTPESILRCEQAGARAFLAKPVVATRLLEVLADVAANTRAEP--GIQSVPALPVG    781
543223      VLSADVTPEAIQRCTQAGAHAFLAKPVVAVRLLDTLAEIASNAQLRTMAAPVVRPAAPVQ    614
533516      VLSADVTPEAIQRCTQAGAHAFLAKPVVAVRLLDTLAEIASNAQLKTMAAPVVRPAAPLQ    650
            *********:* ** ****:*********.***:.**::*:*:: .   .    ** *:

543220      DGVLDTAVLDELASLGMGDGFERKFIDQCLADVDASMGQLQGCGERQAWEDFREHAHALR    841
543211      DGVLDTAVLDELASLGMGDGFERKFIDQCLADVDASMGQLQGCGERQAWEDFREHAHALR    841
543223      DGVLDSSVLDELASLGMGEGFEREFIRQCLEDAASCMGKAEQAGETAQWEVFREQSHAIK    674
533516      DGVLDSSVLDELASLGMGEGFEREFIRQCLEDVASCMGKAEQAGEAAHWDVFREQSHAIK    710
            *****::***********:****:** *** *. :.**: : .**   *: ***::**::

543220      GVASNLGLVQVAASSGEIMRMADWQLKGEWSSRLGNLATALRNGRNALAARSAGAPHRDD    901
543211      GVASNLGLVQVAASSGEIMRMADWQLKGEWSSRLGNLATALRNGRNALAARSAGAPHRDD    901
543223      GVASNLGLMRASNRAGELMRMADWQLKAEWRVRLGVLQDAIKEGRRALDARAERRLRGAA    734
533516      GVASNLGLMRASNRAGELMRMADWQLKTEWRTRLGILQEAIKEGRRALDARAERRMRGAA    770
            ********::.:  :**:********* **  *** *  *:::**.** **:    :

543220      SERSPS       907
543211      SERSPS       907
543223      EDGE--       738
533516      DDGEAR       776
            .: .
```

Supplementary figure 2.

Alignment of the RPF sensor protein. The four sequences represent the different gene clusters present in our strain collection.
